# Mental Nurses and War Hospital Work

**Published:** 1916-08-26

**Authors:** 


*To the Editor of* The Hospital.

### Mental Nurses and War Hospital Work.

*To the Editor of* The Hospital.

Sir,—Why are certificated mental nurses refused positions even as staff nurses in auxiliary or military hospitals? In every paper one reads of the great dearth of trained nurses. Yet how many of trained nurses who hold the medico-physiological certificate have been refused in their application to nurse our wounded since war broke out in August 1914. The nursing authorities connected with the above hospitals do not seem to grasp what training a mental nurse receives.

A nurse trained in a hospital for mental diseases receives a training which befits her for medical, surgical, and phthsisical, as well as mental, nursing. Unless she has a good practical knowledge of the three former sections of nursing she can in no way gain the M.P. certificate. A sister or charge nurse of a mental ward has generally thirty to forty patients, the greater number being in an infirm condition, unable to help themselves in any way, often being in a condition worse than childhood. A mental nurse has her work laid out, yet a bed-sore is an unheard-of thing in her ward. Why? Because a mental nurse attends to her patients as a gardener tends to his master's most valuable plants. You say she has no surgical experience. What about the many suicidal patients which are admitted with their throats severely cut? Are their wounds left unattended, or a surgical nurse called in. No; every nurse in each ward can dress those wounds. Even a probationer in her first three months can dress wounds, as it is necessary for every nurse on her entrance into a mental hospital to acquire the knowledge of first-aid at any rate.

Then what of the epileptics often admitted scalded from head to foot, or severely burned? Are their wounds unattended? What about general paralysis: patients who have been left too long under the care of incompetent persons at home, and are admitted with elbows, knees, buttocks, and shoulders all in a disgraceful and painful condition?

What surgical experience is gained there? What precautions have to be taken? Yes; it would be beneficial at the present day if some officials of auxiliary and military hospitals would even take a day in a mental hospital, and perhaps they would recognise then that certificated mental nurses were qualified to help in the nursing of our wounded. They are nurses in the true sense of the word; also they must be good disciplinarians else they could not manage to have the peace and order which is found in the well-ordered wards of the present mental hospitals; for patients there are not responsible for their actions.

Many hospitals at present containing our wounded refuse certificated mental nurses, yet allow V.A.D.s of all ages to dress wounds, attend to the giving out of medicines, and pneumonia cases in a critical condition. Also there are many hospitals for nervous complaints for our wounded which are staffed, in fact are in command, by persons who have had no training in that respect. Why are they allowed to hold such responsible positions when nurses holding the M.P. certificate are more efficient? A mental nurse with a fair amount of surgical experience would never dream of applying or take the post of theatre sister in a general hospital. Yet is not the nursing of brain disorder more serious?

Surely the medical superintendents of mental hospitals or the members of the Medico-Physiological Association shall agree that their nurses holding the M.P. certificate are qualified to take a staff nurse's position in any of the home hospitals to-day; in fact, many hospitals to-day are staffed with less efficient persons, who hold only a good private social position. Also I am sure mental nurses would be very useful for active service, as their training makes them ready for all emergencies, and many of our brave lads would perhaps be saved from lengthy illnesses if at the beginning they received the care from nurses who understood the nursing of mental disorders.

Trusting that some reader or readers shall take up this question, as there are numbers of good, conscientious nurses who are willing to give their services to their country, but are refused with the same old answer. Their training and certificate stands for nothing, and yet any nurse can be a general trained nurse, but it is not every nurse who can be a mental nurse. Thus, why are they refused to help to-day when every trained nurse is required?
Justice.

[We deal with the questions asked in this letter and other points on page 493.—Ed. The Hospital.]

